# Chimeric antigen receptor-T cell therapy-related cardiotoxicity in adults and children cancer patients: A clinical appraisal

**DOI:** 10.3389/fcvm.2023.1090103

**Published:** 2023-02-21

**Authors:** Massimiliano Camilli, Luca Maggio, Lorenzo Tinti, Priscilla Lamendola, Gaetano Antonio Lanza, Filippo Crea, Antonella Lombardo

**Affiliations:** ^1^Department of Cardiovascular and Pulmonary Sciences, Catholic University of the Sacred Heart, Rome, Italy; ^2^Department of Cardiovascular Medicine, Fondazione Policlinico Universitario A. Gemelli IRCCS, Rome, Italy

**Keywords:** chimeric antigen receptor T-cell therapy, cardiotoxicity, heart failure, cardio-oncology, cardio-immunology

## Abstract

Chimeric antigen receptor-T (CAR-T) cells therapies represent an innovative immunological treatment for patients suffering from advanced and refractory onco-hematological malignancies. The infusion of engineered T-cells, exposing chimeric receptors on the cell surface, leads to an immune response against the tumor cells. However, data from clinical trials and observational studies showed the occurrence of a constellation of adverse events related to CAR-T cells infusion, ranging from mild effects to life-threatening organ-specific complications. In particular, CAR-T cell-related cardiovascular toxicities represent an emerging group of adverse events observed in these patients, correlated with increased morbidity and mortality. Mechanisms involved are still under investigation, although the aberrant inflammatory activation observed in cytokine release syndrome (CRS) seems to play a pivotal role. The most frequently reported cardiac events, observed both in adults and in the pediatric population, are represented by hypotension, arrhythmias and left ventricular systolic dysfunction, sometimes associated with overt heart failure. Therefore, there is an increasing need to understand the pathophysiological basis of cardiotoxicity and risk factors related to its development, in order to identify most vulnerable patients requiring a close cardiological monitoring and long-term follow-up. This review aims at highlighting CAR-T cell-related cardiovascular complications and clarifying the pathogenetic mechanisms coming at play. Moreover, we will shed light on surveillance strategies and cardiotoxicity management protocols, as well as on future research perspectives in this expanding field.

## 1. Introduction

In the recent years, immune-related therapies have revolutionized the field of onco-hematology, gaining a primary role in the treatment of solid and liquid cancers associated with poor prognosis (1).

Chimeric antigen receptor T (CAR-T) cells administration represents a novel paradigm of cancer management, consisting in the infusion of “engineered” immune cells able to elicit a response against the tumor (2). The biological foundations of this treatment are layed on the development of *in vitro* mature T-cell receptor (TCR)-lymphocytes, created to recognize tumor cells antigens (3). These chimeric receptors are composed by four domains: the extracellular antigen-binding portion, tailored to recognize the specific tumor target, an hinge point, the transmembrane domain and the signaling domain, responsible for signal transduction into the cell and linked with different other co-stimulatory molecules (4). The antigen-binding domain, constituted by an antibody-specific fragment obtained from monoclonal antibodies, leads to the recognition of the target tumor antigen, subsequently resulting in a major histocompatibility complex (MHC)-independent activation of the engineered T cells antitumor function (5, 6). In order to enhance the efficacy of CAR-T cells, a chemotherapeutic scheme containing fludarabine/cyclophosphamide is generally co-administrated. This conditioning regimen determines different biological effects, such as lymphodepletion, eradication of immunosuppressive cells (regulatory T cells and myeloid-derived suppressor cells), modulation of tumor microenvironment and increased expansion and persistence of CAR-T cells (7–10).

Over the years, numerous studies have been carried out with the aim of improving T cells clonal expansion and cytokine secretion. The first CAR-T cell therapy to be approved in 2017 was Axicabtagene Ciloleucel, developed to treat adults with relapsed or refractory (R-R) large B-cell lymphoma expressing membrane antigen cluster domain (CD)19 (11). Approvement came after the ZUMA-1 trial, a multicenter phase 2 study, that reported in the 101 patients finally treated, an objective response rate of 82 and 54% of complete remission rate (12). Tisagenlecleucel was instead, distributed after the results of the ELIANA trial, which reported an overall remission in 81% of the 75 patients enrolled within 3 months (13, 14). The non-CD19 cell therapy firstly approved was Idecabtagene Vicleucel, created against B-cell maturation antigen (BCMA): this demonstrated an encouraging response rate of 73% in a phase II study in patients with R-R multiple myeloma (15).

Thanks to the success obtained in B-cell malignancies, new trials have been launched with the aim of replicating the same results in other tumors, such as non-Hodgkin lymphoma (NCT02315612) and chronic lymphocytic leukemia (NCT02194374), or otherwise solid neoplasms like hepatocellular carcinoma (NCT02541370), breast cancer (NCT00673829) or pancreatic cancer (NCT02706782) (5).

Similarly to other oncological targeted therapies, CAR-T cells demonstrated in clinical trials to be associated with several adverse events, sometimes fatal. In particular, available evidences, including prospective and retrospective reports, highlighted a correlation between CAR-T cell therapy and cardiovascular (CV) adverse complications, both in children and adults, significantly affecting morbidity and mortality (12, 13, 15–24). Given the increasing number of patients who will benefit from this therapy in the near future, it is expected that CAR-T cell therapy-related cardiotoxicity will become a clinical problem not uncommon to cardiologists. Therefore, the aim of this review is to provide a general picture of CAR-T cell therapy CV effects, briefly describing their pathophysiological mechanisms and clinical implications. We will make a distinction between adult and pediatric population, provide practical management/surveillance advises and shed light on future research perspectives in this field.

## 2. Pathogenesis of CAR-T cell therapy CV toxicity

The spread of CAR-T cell therapy in the hemato-oncological population has led to the identification of novel forms of toxicity, both systemic and organ-specific (25). The most common manifestation of CAR-T cell-related toxicity is the cytokine release syndrome (CRS), characterized by a proinflammatory cytokine “storm,” subsequent to the infusion of cells and their interaction with the tumor microenvironment (26). In the 2018 American Society for Transplantation and Cellular Therapy (ASTCT) consensus, CRS is defined as a supraphysiologic response following any immune therapy that results in the activation or engagement of endogenous or infused T cells and/or other immune effector cells. In CRS clinical presentation, symptoms can be progressive and may include fever (mostly present at onset), hypotension, hypoxia, capillary leak syndrome and life-threatening organ dysfunctions (27).

In Lee et al. (28) proposed a revised grading system of CRS: in grade 1 symptoms are not life threatening (fever, nausea, fatigue, headache, myalgias) and require symptomatic treatment only; in grade 2 symptoms require and respond to moderate intervention, such as hypotension responsive to fluids or low dose of one vasopressor or Grade 2 organ toxicity, with oxygen requirement < 40% FiO2. Grade 3 symptoms require an aggressive intervention, such as hypotension requiring high dose or multiple vasopressors, Grade 3 organ toxicity or grade 4 transaminitis, with oxygen requirement > 40% FiO2. In Grade 4, symptoms are life-threatening, with requirement for ventilator support or Grade 4 organ toxicity (excluding transaminitis); lastly, grade 5 relates instead to death (28, 29).

The basis of these manifestations is complex: Morris et al. (30) proposed a five phases process of CRS pathophysiology. The first relates to the interaction between CAR-T cells and tumor site, with the recognition of antigen-expressing target cells; gradually the proliferation of CAR-T cells and *in situ* cytokine production by both CAR-T cells and cellular components of the tumor microenvironment occur. The proliferation of CAR-T cells, together with increased cytokine levels, leads to a systemic inflammatory response, associated with endothelial injury and tissue capillary leakage. Progressively, the transmigration of cytokines, CAR-T cells and immune system cellular effectors into the central nervous system, causes a breakdown of the blood–brain barrier. At last, the eradication of the tumor and the inactivation of the immune response, results in decreased cytokine levels and systemic inflammatory response. Therefore, the interaction between CAR-T cells and tumor microenvironment, and the associated up-regulation of proinflammatory cytokine secretion into the bloodstream, represent a crucial phase in the genesis of CRS (31).

Different studies on murine models highlighted the main role of *in situ* monocyte/macrophage cells in the secretion of pro-inflammatory cytokines, such as interleukin (IL)-6 and IL-1, and the correlation between their blood levels and CRS mortality (32, 33). In these reports, the blockade of IL-6 and IL-1 signaling pathways in humanized murine models caused downregulation of proinflammatory cytokine secretion and resolution of most CRS clinical manifestations. As a result, various consecutive clinical studies demonstrated the role of anti-IL-6 monoclonal antibody tocilizumab as first-line therapy in CRS management; ongoing clinical trials on IL-1 antagonist Anakinra aim to prove its role as an emergent therapeutic option (NCT04148430) (32, 34, 35).

Other pro-inflammatory soluble effectors revealed to play a pivotal role in the CRS pathogenesis, such as Granulocyte-Macrophage Colony Stimulating Factor (GM CSF) and Tumor Necrosis Factor (TNF)-alpha The blockade of their signaling pathways on murine models has also been shown to be associated with reduced IL-6 levels and CRS manifestations, without interfering with the antitumor effects of CAR-T cells (36, 37).

That said, although mechanisms involved in the pathogenesis of CV toxicity may be numerous and are still under investigation, the abnormal proinflammatory cytokine release related to the occurrence of CRS is one of the most recognized mediators so far.

The resemblance of CAR-T cell related cardiac dysfunction with cardiomyopathies observed during sepsis, has suggested the recognition of the elevated levels of IL-6 as a common substrate of myocardial depression (38). Actually, in sepsis-related cardiomyopathy, the elevated levels of proinflammatory cytokines leads to microvascular dysfunction with capillary permeability, mitochondrial dysfunction and altered intracellular calcium metabolism, that are responsible for myocardial inflammation and impaired perfusion (39). As exposed afterward, alterations in myocardial performance, observed in patients undergoing CAR-T cell infusion, are most commonly related to higher grades of CRS, highlighting the close relationship between cardiomyocyte damage and inflammatory response.

Another peculiar mechanism of CV toxicity was observed by Linette et al. (40), that firstly described two cases of fatal myocarditis and cardiogenic shock occurred as a result of cross-reactivity of engineered T cells expressing an affinity-enhanced receptor against MAGE-A3 (melanoma-associated antigen-3), which cross-reacted with titin, a myocardial protein. Of note, these manifestations were observed with an old generation of engineered T-cells therapy, and to date, there are no reports of cardiac toxicity through cross-reactivity with the new approved CAR-T cells, although research in this field is needed.

In these pathogenetic considerations, it should be acknowledged that patients undergoing CAR-T cell administration have been already exposed to numerous potentially cardiotoxic chemotherapy lines, influencing the susceptibility to myocardial dysfunction (41).

Table 1 summarizes available studies in the adult and pediatric population.

**TABLE 1:** Summary of cardiovascular adverse events reported in studies enrolling adult and pediatric patients.

Study	Disease and patient population in safety analysis	Grade ≥ 3 CRS	Hypotension requiring vasopressor support or shock	Reduced EF	Tachycardia	Cardiac arrest	Other cardiac adverse events
**Adult population**							
Neelapu et al. (12) (ZUMA-1) phase II clinical trial	LBCL (*n* = 101)	13 (12.9%) (Lee criteria) (28)	14 (13.9%)	–	39 (39.4%)	1 (<1%)	Cardiac death: 1 (<1%)
Schuster et al. (44) (JULIET) phase II clinical trial	LBCL (*n* = 111)	24 (21.6%) (Penn criteria) (60)	10 (9%)	–	12 (10.8%)	–	–
Wang et al. (22) (ZUMA-2) phase II clinical trial	MCL (*n* = 68)	10 (14.7%) (Lee criteria) (28)	15 (22.1%)	–	21 (30.9%)	–	–
Alvi et al. (21) retrospective	NHL, MM (*n* = 137)	6 (4.4%) (Lee criteria) (28)	–	8/29 (27.6%)*,¥	–	3 (2.2%)	Cardiac death: 6 (4.4%) HF: 6 (4.4%) arrhythmia: 5 (3.6%) elevated troponin: 29/53 (54.7%)^∘^
Ganatra et al. (50) retrospective	NHL, B-ALL, PML (*n* = 187)	10 (5.3%) (Lee criteria) (28)	14 (7.5%)	12/116 (10.3%)^, ¥	–	–	Cardiac death: 3 (1.6%)
Lefebvre et al. (17) retrospective	NHL, B-ALL, CLL (*n* = 145)	–	33 (22.7%)	–	–	1 (0.7%)	Cardiac death: 2 (1.4%) HF: 21 (14.5%) Arrhythmia: 13 (8.9%) ACS: 2 (1.3%)
Munshi et al. (15) (NCT03361748) phase II clinical trial	MM (*n* = 128)	7 (5.5%) (Lee criteria) (28)	1 (<1%)	–	–	–	–
Goldman et al. (19) retrospective	Various (*n* = 2657)	–	–	69 (2.6%)^∘∘^	–	–	Arrhythmia: 74 (2.8%) pericardial disease: 11 (0.4%) VTE: 28 (1.6%) cardiogenic shock: 49 (1.8%)
**Pediatric and young adult population**							
Maude et al. (52) phase I-IIa clinical trial	B-ALL (*n* = 30)^∧∧^	8 (27%)	8 (27%)	–	–	–	Severe coagulopathy: 3 (10%)
Lee et al. (51) phase I clinical trial	B-ALL (*n* = 25)	6 (32%) (Lee criteria) (51)	4 (22%)	1 (5%)¬	–	1 (5%)	QT prolongation: 1 (5%)
Fitzgerald et al. (18) retrospective	B-ALL (*n* = 39)	18 (46%) (Porter criteria) (61)	13 (33%)	1 (2%)∞	–	–	–
Maude et al. (13) (ELIANA) phase II clinical trial	B-ALL (*n* = 75)	25 (46%) (Penn criteria) (60)	13 (17%)	3 (4%)**	3 (4%)	3 (4%)	HF: 2 (2.7%)
Burstein et al. (54) retrospective	B-ALL, NHL, T-ALL, APL (*n* = 98)	24 (24%) (Penn criteria) (60)	24 (24%)	10 (10%)±	–	0	ST segment changes: 6 (6%)
Shalabi et al. (23) retrospective	B-ALL, NHL (*n* = 52)	9 (17%) (Lee criteria) (27, 28)	9 (24.3%)	6 (11.5%)⊥	36 (69.2%)	1 (2.7%)	

APL, acute promyelocytic leukemia; B-ALL, B-cell acute lymphoblastic leukemia; ACS, acute coronary syndrome; CLL, chronic lymphocytic leukemia; CRS, cytokine release syndrome; EF, ejection fraction; HF, hearth failure; LBCL, large B-cell lymphoma; MCL, mantle cell lymphoma; MM, multiple myeloma; NHL, non-Hodgkin lymphoma; PML, primary mediastinal large B-cell lymphoma; T-ALL, T cell-acute lymphoblastic leukemia; VTE, venous thromboembolic events.

*Considered only patients with echocardiographic data pre- and post-CAR-T.

^∘^Considered only patients with post-CAR-T troponin level assessment.

^∧^Considered only patients with serial echocardiogram.

^∧∧^Considered a sample size of 25 children and 5 adults.

¥Reduced EF: reduction in LVEF > 10% from baseline to <50%.

^∘∘^Left ventricular systolic dysfunction according to MedDRA version 22.1 (62).

±Reduced EF: decrease of ≥10% in ejection fraction or ≥5% in shortening fraction compared with baseline or ejection fraction < 55% or shortening fraction < 28% in those with previously normal systolic function (54).

⊥Reduced EF: decrease of >10% absolute decrease in LVEF compared with baseline or new-onset LVEF < 50% (23).

¬LV systolic dysfunction according to Common Terminology Criteria for Adverse Events v4.02 (63).

∞LV systolic dysfunction according to Goldstein et al. (64).

**LV systolic dysfunction according to Common Terminology Criteria for Adverse Events v4.03 (63).

## 3. CAR-T cell therapy cardiotoxicity in adult patients

Although patients with recent or previous cardiovascular events were excluded from main clinical trials leading to the approval of CAR-T cell therapies, in real-world observational data a high percentage of subjects undergoing this therapy experienced CV side effects, that have been associated with significant morbidity and mortality (41). Increasing pharmacovigilance data about patients treated with CAR-T cell therapies are now available.

A wide variety of cardiac adverse events has been reported in adults, including hypotension (in some cases life-threatening requiring vasopressor support), arrhythmias, left ventricular systolic dysfunction, myocardial injury, ST-segment changes on the electrocardiogram (ECG) and rarely cardiac death (42). Most of cardiac manifestations reported, in particular hypotension and reflex tachycardia, can arise as cardiovascular issues or mostly as consequences of CRS (43), so it may result difficult to regard them as pure cardiotoxic events.

Goldman et al. (19) reported data on 2,657 patients exposed to CAR-T cell therapy (65% treated with Axicabtagene and 35% with Tisagenlecleucel). The treatment was associated with hypotension (10.8%), tachyarrhythmias (2.8%, among which atrial fibrillation was the most frequent), cardiomyopathy (2.6%), cardiogenic shock (1.8%) and pericardial disease (0.4%).

When comparing CAR-T products, Axicabtagene was associated with higher reporting of CRS (59% vs 47%), tachyarrhythmias (3.4% vs 1.7%), and VTE (1.6% vs 0.7%). Any grade CRS was reported in 55% of studied population and the authors further focused on the overlap between CRS and cardiovascular events. Concurrent CRS was reported in 78% of hypotension cases, 79% of tachyarrhythmia, 65% of cardiogenic shocks, 51% of cardiomyopathy cases and with 64% of pericardial manifestations (19).

Analogously, Salem et al. (20) described the treatment effects on 1,921 subjects, among which 13.3% documented similar cardiac events, all of which occurred in association with grade ≥ 2 CRS. Among patients suffering from cardiac events, 57% were treated with Axicabtagene and 43% were treated with Tisagenlecleucel (20).

As far as clinical trials on CAR-T cell therapies are concerned, in ZUMA-1, JULIET, ZUMA-2 and NCT03361748, hypotension of any grade was described in 16–59% of the analyzed population, with up to 22% of these requiring vasopressor support. Another cardiac adverse event frequently reported was tachycardia (11–39%). As well, in ZUMA-1 trial, a case of cardiac arrest occurred in a patient with grade 5 CRS (12, 15, 22, 44). Nevertheless, the 2-year follow-up data of the same trial showed development of hypertension in 16% of patients (45).

In depth, Neelapu et al. (12) and Wang et al. (22) reported data about the correlation between CRS and cardiovascular events: in ZUMA-1 trial, 54% of tachycardia cases, 68% of any grade hypotension and 64% of ≥3 grade hypotension were associated with CRS, while in ZUMA-2 all cases of hypotension and 76% of tachycardia occurred in the context of CRS.

Smaller phase I clinical trials reported grade ≥ 3 hypotension in 20–37% and left ventricular dysfunction in 10% of treated patients (46–49).

As mentioned, all reported clinical trials excluded subjects with significant pre-existing cardiovascular disease (e.g., heart failure) (12, 15, 22, 44) and they did not focus on the correlation between cardiovascular risk factors and safety endpoints.

Besides clinical trials, real-world, observational studies included, even if marginally, patients with baseline significant cardiac disease (17, 21), and made an attempt to evaluate the role of pre-existing CV risk factors and cardiac biomarkers to predict the occurrence of cardiotoxicity. In a retrospective study of 137 patients treated with CAR-T therapy, Alvi et al. (21) described 17 cases of cardiovascular complications (12%), including 6 cases of cardiovascular death, 6 cases of HF and 5 cases of supraventricular arrhythmia. Of interest, all these events occurred in patients developing high-grade CRS (≥2), and among this population, 69% were treated with Axicabtagene and 31% with other investigational CAR-T.

Furthermore, in this study, the authors evaluated the potential role of troponin in predicting cardiotoxicity. Post-CAR-T cell infusion troponin levels were measured, witnessing that CV event rate for patients with elevated troponin was 55%, compared to 4% in patients without elevation.

As for cardiovascular events, also troponin elevation was closely related to CRS grading (reported in 77% of patients with ≥2 CRS grade vs 22% of patients with lower grade CRS). Among subjects with troponin release, 45% were treated with Axicabtagene and 55% with investigational CAR-T.

When pre- and post-CAR-T cell therapy left ventricular ejection fraction (LVEF) assessment was available, a significant reduction in LVEF was observed in 28% of cases. As mentioned, all these patients had also elevated troponin levels at blood samples (21).

Another retrospective study conducted by Lefebvre et al. (17) evaluated the correlation between major adverse cardiac events (MACE) and population baseline characteristics. In 145 patients, it was reported a total of 41 MACEs. Some pre-CAR-T cell therapy features, as the use of statins, insulin and aspirin, as well as higher baseline creatinine levels, were each associated with MACE, likely reflecting patients with high cardiovascular risk profile and pre-existing comorbidities. The grade of CRS (27) was also defined as an independent predictor of MACE. Baseline LVEF was not associated with MACE in this population, unlike some diastolic parameters, such as elevated E/e’ ratio and left atrial volume could represent valuable predictors (17).

Ganatra et al. (50) otherwise focused on development of cardiomyopathy following CAR-T cell therapy in a sample size of 116 patients who underwent serial echocardiograms. Their results demonstrated that 12 patients (about 10% of the population) developed new-onset or worsening cardiomyopathy, nearly always (11/12) in association with grade ≥ 2 CRS. Again, baseline features such as hyperlipidemia or older age, history of coronary artery disease and use of beta blockers and renin-angiotensin inhibitors therapy, appeared to increase the risk of developing cardiomyopathy. Among those patients (*n* = 12) with cardiomyopathy, 6 had a complete recovery of LVEF, 3 had a partial recovery and 3 died (50). In this report, Axicabtagene was the main treatment investigated (used in 94% of the patients) and it was associated with the majority of cases of new-onset or worsening cardiomyopathy (92% vs 8% associated with Tisagenlecleucel).

Overall, in retrospective studies, the timing from CAR-T infusion to reported cardiac events ranged from a median of 5–21 days (42). This suggests that in adult patients, cardiotoxicity could be closely related to high grade CRS onset and subsequent resolution.

Data regarding the CV effects of CAR-T cell therapy in adults are illustrated in Table 1.

## 4. CAR-T cell therapy cardiotoxicity in the pediatric and young adults population

Thanks to the available data, we are now more aware of the prevalence and impact on morbidity and mortality of CAR-T cell-related CV toxicity in the pediatric and young adult population.

In depth, Lee et al. (51) led a phase I trial enrolling children and young adults with R-R acute lymphoblastic leukemia or non-Hodgkin lymphoma; in a cohort of 21 subjects, 4 (22%) developed severe hypotension requiring vasopressor support, 1 had QT prolongation and 1 hypertension. Furthermore, one patient was successfully resuscitated after cardiac arrest during a severe CRS, associated with a drop in his cardiac ejection fraction from a baseline of 65% to less than 25% (51). In a comparable limited cohort of 30 children and adults with relapsed acute lymphoblastic leukemia (ALL), 27% required vasopressor support for hypotension and 3 subjects presented bleeding due to severe coagulopathy (52).

The ELIANA and ENSIGN phase-2 clinical trials enrolled CD19 + relapsed or refractory B-cell ALL patients, exposed to Tisagenlecleucel, an anti-CD19 CAR T-cell therapy (13, 53). In the face of impressive clinical benefits and hematological disease response, they demonstrated that CV complications were predominantly seen in the first 8 weeks post-infusion, especially during CRS, and were mostly reversible. A pooled analysis from these trials (ELIANA: *n* = 79; ENSIGN: *n* = 58) reported that in a total analyzed population of 137 patients, 31% presented cardiac complications, 7% of grade 3 or 4. The most common events were arrhythmias (29%); five patients (4%) developed left ventricular cardiac dysfunction during CRS, which regressed in all but 2 patients, who died due to disease progression. Hypotension occurred in 24% of the patients, 19% of grade 3 or 4 requiring intervention or vasopressor support; interestingly, hypotension was always related with CRS (53). In the ELIANA cohort, 3 (4%) subjects developed LV dysfunction, 2 patients presenting with symptomatic heart failure, and all had cardiac arrest (13).

As regards to retrospective studies, data seem more homogeneous. Fitzgerald et al. (18) reported, in 39 patients receiving CAR-T cells infusion for R-R ALL, that 36% showed CV complications, 13 with fluid refractory shock requiring the infusion of α-agonist, 10 necessitating more than one vasopressor due to catecholamine resistance and 1 supported with milrinone because of the development of diminished left ventricular systolic function (18). CV complications generally occurred within the 5 days after cell infusion, hypotension was always preceded by fever and 96% of the patients with CV complications also had CRS, with 18% CRS grade 3 and 28% CRS grade 4 (18).

Shalabi et al. (23) retrospectively revised the cases of 52 pediatric patients affected by hematological malignancies; nine of them (17%) developed hypotension requiring vasopressor support. Of the 37 patients with CRS, 6 (16%) presented a post-infusion echocardiogram showing cardiac dysfunction, among whom 4 had grade 3–4 CRS. Of importance, all cases with cardiac dysfunction and HF recovered within 3 months. Moreover, it was noted a higher likelihood of reduced cardiac performance in patients with early and severe CRS, as well as in those receiving tocilizumab. As acknowledged, none of the cases without CRS experienced CV toxicity (23).

In another report including 98 subjects and aimed at identifying predictors of CV toxicity (in particular of hypotension requiring vasopressor support), 24 experienced shock and 10 (41%) of them concomitantly developed cardiac dysfunction. Five subjects with cardiac dysfunction required inotropic support with milrinone and 6 of these needed pharmacological escalation. In accordance with data from other published retrospective studies, all patients affected by CV toxicity had grade 3–4 CRS and those with impaired systolic function recovered by 6 months of follow-up; nevertheless, none of the cardiac events contributed to mortality (54).

In summary, from all the studies available, it seems that cardiac toxicity associated with CAR-T in the pediatric and young adult population is closely associated with CRS and is largely self-limited, with most patients recovering cardiac function back to pre-treatment baseline values within few weeks from exposition (23, 54).

Nevertheless, the majority of data on determinants of cardiotoxicity come from retrospective studies. In pediatric and young adult patients, the risk of developing severe hypotension requiring vasopressor support appears to be associated with high disease burden (>25% of blast on bone marrow biopsy), pre-treatment cardiac dysfunction (lower baseline ejection fraction, lower GLS or diastolic dysfunction), high-grade CRS (3-4), and delays in the administration of tocilizumab for CRS. Pre-existing cardiomyopathy and higher anthracycline dose did not appear to be associated with hypotension-requiring inotropic support in previous reports (42, 54).

There is still little knowledge regarding the predictive role of cardiac biomarkers, such as ultra-sensitive troponins and Brain Natriuretic Peptides (BNP) or N-terminal pro-BNP (NTpro-BNP). In the Shalabi et al. (23) cohort, 13 patients underwent troponin levels dosage during CRS: 4 cases resulted with abnormal troponin values, all of them associated with cardiac dysfunction quantified by decreased LVEF; NTpro-BNP was assessed only in 7 patients and proved to be elevated during CRS, with the highest values observed in two patients with cardiac impairment developed during CRS (23). Because of the scarceness of investigations, it is not possible to establish a precise role for cardiac biomarkers in patients undergoing CAR-T cell therapy, including their usefulness for patients’ surveillance.

## 5. Monitoring and management of CV complications in patients undergoing CAR-T cell therapy

Considering the burden of CV complications occurring during CAR-T cells infusions, the latest 2022 ESC cardio-oncology guidelines recommend to perform a baseline cardiological evaluation, including electrocardiogram, natriuretic peptides, troponins and transthoracic echocardiogram, in particular in subjects with pre-existing CV conditions (41). However, it should be acknowledged, due to the patient heterogeneity and small sample sizes of available studies, that these recommendations only rely on expert consensus and not on high-quality evidences.

During infusion, frequent vital signs assessment and arrhythmias monitoring through telemetry should be performed. Volume resuscitation with intravenous fluid represents the first-line therapy for hypotension and shock related to CRS (42). For resistant cases, vasopressors (e.g., adrenaline, noradrenaline, vasopressin) should be considered (26, 55, 56). However, other causes of hypotension and shock, such as severe infections, cardiac tamponade, pulmonary embolism or acute cardiac dysfunction, should be ruled out.

The occurrence of signs of high-grade CRS should be promptly followed by complete cardiac evaluation, including electrocardiogram, biomarkers and echocardiogram (57).

Tocilizumab is an anti-IL-6-receptor antagonist that is approved to manage severe CRS and may also mitigate CV toxicity of CAR T-cells (58, 59). Tocilizumab should hence be used in subjects with CRS grade 2 or higher and with high suspicion of CV complications: troponin elevation may help identifying candidates experiencing CRS for early tocilizumab administration (21). Nevertheless, prospective investigations are needed to define the optimal management strategy for CAR T-cell related cardiotoxicity.

**FIGURE 1:**
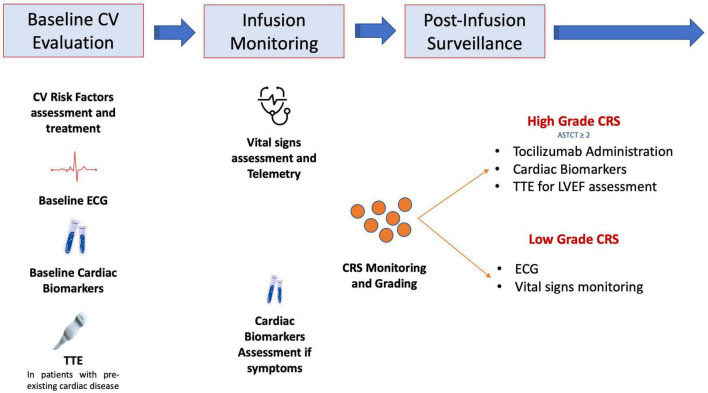
Proposed diagnostic work-up and management strategy in patients undergoing chimeric antigen receptor (CAR)-T cell treatment and with suspected cardiovascular toxicity. ASTCT, American Society for Transplantation and Cellular Therapy; CRS, cytokine release syndrome; CV, cardiovascular; ECG, electrocardiogram; TTE, transthoracic echocardiography.

Figure 1 shows a proposed diagnostic work-up and management strategy of CV complications in patients exposed to CAR-T cell therapy.

## 6. Future perspectives

Mechanisms underlying CV events in patients undergoing CAR-T cell infusion are poorly understood; basic mechanistic studies are therefore needed in order to investigate possible myocardial damage pathways, which may occur regardless of cytokine release and CRS. For example, measurement of soluble cardio-depressant factors, commonly found in patients with septic cardiomyopathy, could help identifying novel mechanisms of cardiac failure. On the other hand, prospective imaging studies, using non-invasive advanced metrics of myocardial function (e.g., strain imaging with speckle tracking) are also necessary. At last, a multidisciplinary approach to the management of patients on CAR-T cells is needed to optimize outcomes and to ensure a comprehensive care of these patients.

## 7. Conclusion

Chimeric antigen receptor T-cell immunotherapy has recently changed the scenario of cancer treatment, with expanding indications also for solid tumors. Therefore, the awareness of its possible complications is of utmost importance, in order to early identify and treat these manifestations. However, at this point in time, preventive measures, pharmacological management and surveillance strategies remain poorly understood and data available rely on retrospective reports. Dedicated prospective clinical studies, including large cohorts of patients, are hence warranted in order to clarify the CV involvement pathogenesis, the use of imaging and serum biomarkers in events identification and treatment opportunities.

## Author contributions

MC, LM, and LT wrote the manuscript, prepared the figure and table, and edited the manuscript. MC, PL, GL, FC, and AL critically reviewed the manuscript. All authors contributed to the article and approved the submitted version.
